# Cell-cycle and apoptosis related and proteomics-based signaling pathways of human hepatoma Huh-7 cells treated by three currently used multi-RTK inhibitors

**DOI:** 10.3389/fphar.2022.944893

**Published:** 2022-08-22

**Authors:** Xuejiao Ren, Qingning Zhang, Wenyan Guo, Lan Wang, Tao Wu, Wei Zhang, Ming Liu, Dezhi Kong

**Affiliations:** ^1^ Department of Radiotherapy, Third Hospital of Hebei Medical University, Shijiazhuang, China; ^2^ Department of Radiotherapy, Fourth Hospital of Hebei Medical University, Shijiazhuang, China; ^3^ Department of Pharmacology of Chinese Materia Medica, School of Chinese Integrative Medicine, Hebei Medical University, Shijiazhuang, China

**Keywords:** cell cycle, cell apoptosis, proteomics, signal pathway, sorafenib, lenvatinib, regorafenib, Huh-7 cells

## Abstract

Sorafenib, lenvatinib and regorafenib, the multi-RTK inhibitors with potent anti-angiogenesis effects, are currently therapeutic drugs generally recommended for the patients with advanced hepatocellular carcinoma (HCC). To date, however, there have been no published studies on the mechanism underling differential effects of the three drugs on HCC cell proliferation, and the proteomic analysis in HCC cell lines treated by regorafenib or lenvatinib. The present study for the first time performed a direct comparison of the cell cycle arrest and apoptosis induction in the Huh-7 cells caused by sorafenib, regorafenib and lenvatinib at respective IC_50_ using flow cytometry technique, as well as their pharmacological interventions for influencing whole cell proteomics using tandem mass tag-based peptide-labeling coupled with the nLC-HRMS technique. Sorafenib, regorafenib and lenvatinib at respective IC_50_ drove the remaining surviving Huh-7 cells into a G_0_/G_1_ arrest, but lenvatinib and regorafenib were much more effective than sorafenib. Lenvatinib produced a much stronger induction of Huh-7 cells into early apoptosis than sorafenib and regorafenib, while necrotic cell proportion induced by regorafenib was 2.4 times as large as that by lenvatinib. The proteomic study revealed 419 proteins downregulated commonly by the three drugs at respective IC_50_. KEGG pathway analysis of the downregulated proteins indicated the ranking of top six signaling pathways including the spliceosome, DNA replication, cell cycle, mRNA surveillance, P53 and nucleotide excision repair involved in 33 proteins, all of which were directly related to their pharmacological effects on cell cycle and cell apoptosis. Notably, lenvatinib and regorafenib downregulated the proteins of PCNA, Cyclin B1, BCL-xL, TSP1, BUD31, SF3A1 and Mad2 much more strongly than sorafenib. Moreover, most of the proteins in the P53 signaling pathway were downregulated with lenvatinib and regorafenib by more than 36% at least. In conclusion, lenvatinib and regorafenib have much stronger potency against Huh-7 cell proliferation than sorafenib because of their more potent effects on cell cycle arrest and apoptosis induction. The underling mechanism may be at least due to the 33 downregulated proteins centralizing the signal pathways of cell cycle, p53 and DNA synthesis based on the present proteomics study.

## 1 Introduction

Although global incidence and mortality rates for cancer show a declining trend, those for liver cancer are increasing (Ryerson et al., 2016; Akinyemiju et al., 2017). Since high incidence of Hepatitis B and C virus infection is observed in China, the mortality and incidence rates of liver cancer in China rank third and fourth respectively in all malignant tumors (Chen et al., 2016). Torre et al. (2015) reported that hepatocellular carcinoma (HCC) accounts for 70∼90% of primary liver cancers (Torre et al., 2015). Leone et al. (2021) indicated that HCC development occurs in a liver that is severely compromised by chronic injury or inflammation. Liver transplantation, hepatic resection, radiofrequency ablation, transcatheter arterial chemoembolization, and targeted therapies based on tyrosine protein kinase inhibitors are the most common treatments (Leone et al., 2021). Due to lack of specific HCC symptoms and predictive biomarkers, most HCC patients are diagnosed in their late stages. Molecular targeting therapy is an important treatment option for the patients with advanced HCC (Llovet and Bruix, 2008). Sorafenib, a multi-receptor tyrosine kinase (RTK) inhibitor with potent anti-angiogenesis effects, is the first FDA-approved molecular-targeted agent for the patients with advanced HCC, and National Comprehensive Cancer Network Clinical Practice Guidelines still recommended it as the first-line therapy today (Benson et al., 2019). However, many patients with advanced HCC are resistant to sorafenib during its treatment (Haga et al., 2017). In the last 5 years, other multi-RTK inhibitors including regorafenib and lenvatinib have been approved. Regorafenib, an approved second-line multi-RTK inhibitor, shows survival benefit in patients with HCC who progressed on sorafenib treatment (Bruix et al., 2017). Kudo et al. (2018) indicated that lenvatinib was non-inferior to sorafenib in overall survival for untreated advanced HCC patients in the randomized phase 3 non-inferiority trials, thereby being approved as a multi-RTK inhibitor for the treatment of advanced HCC currently (Kudo et al., 2018). Lenvatinib combined with sorafenib is expected to be a preferred first-line therapy in patients with advanced HCC (Bouattour et al., 2019).

The concentrations of sorafenib, regorafenib and lenvatinib in human blood were reported to be 10 μM, 2 and 0.1 µM, respectively (Strumberg et al., 2005; Nagahama et al., 2019; Taguchi et al., 2020). In 2020, Sasaki et al. compared the inhibitory effects of sorafenib, regorafenib and lenvatinib using human hepatoma HepG2 cells and human hepatoma Huh-7 cells (Sasaki et al., 2020). However, they found that sorafenib at ∼5 μM, regorafenib at ∼2 µM and lenvatinib at ∼10 µM did not affect the proliferation of HepG2 cells. Even though sorafenib, regorafenib and lenvatinib concentration-dependently produced the inhibition of Huh-7 cell proliferation at 1∼20 μM, their maximal inhibition rates were about 60%, 70% and 50%, respectively (Sasaki et al., 2020). They also investigated whether regorafenib or lenvatinib modulates innate immunity including 84 Toll-like receptor-associated genes in human hepatoma cell lines using real-time RT-PCR analysis and found that 14 genes in Huh-7 cells and 12 genes in HepG2 cells were significantly downregulated by regorafenib; however, six genes in Huh-7 cells and one gene in HepG2 cells were downregulated by lenvatinib significantly (Sasaki et al., 2020). They did not see the protein expression levels in their experiments.

Recently, we revealed that HepG2 cells surviving from the attack of high concentration sorafenib were significantly related to the upregulation of 520 proteins, 33 proteins of them were mainly involved in the mitochondrial metabolic pathway, and 12/33 proteins were closely related to the mitochondrial OXPHOS (Bai et al., 2019). Hou et al. evaluated the suppressive effects of dihydroartemisinin in combination with sorafenib on protein expression and reported 229 proteins upregulated by sorafenib alone in HCC cell line HCCLM3 (Hou et al., 2020). However, whole cell proteomics study in HCC cell lines treated by regorafenib or lenvatinib has not been published to date.

In the present study, we first tried to establish the more satisfactory dose-response curves of the cell growth inhibition by sorafenib, regorafenib and lenvatinib in Huh-7 cells to obtain the value of half-maximal inhibitory concentration (IC_50_) for each agent, as close as possible to the reported concentrations in human blood of the three multi-RTK inhibitors. Then, using the Huh-7 cells incubated with sorafenib, regorafenib and lenvatinib at respective IC_50_, we were able to compare whether there were significant differences in the effects on cell cycle and apoptosis among the three inhibitors. Finally, we performed a comparative analysis using the tandem mass tag (TMT) technology to analyze proteomic changes in Huh-7 cells treated with sorafenib, regorafenib and lenvatinib at respective IC_50_, and further revealed the proteomics-based signaling pathways involved in their inhibition of cell cycle and the induction of apoptosis. Clarifying the above problems will help to develop more effective multi-RTK inhibitors and provide new pharmacological knowledge when using the above drugs, which would be conducive to the rational drug use in patients.

## 2 Materials and methods

### 2.1 Chemicals and biological reagents

Sorafenib, regorafenib and lenvatinib were from Cayman Chemical Company (Michigan, United States); DMSO and bicinchoninic acid (BCA) assay were from Solarbio Science and Technology Company (Beijing, China); fetal bovine serum (FBS), Dulbecco’s modified essential media (DMEM) and trypsin-EDTA were from Gibco (California, United States); cell counting kit-8 (CCK-8) was from Dojindo Corporation (Shanghai, China); penicillin-streptomycin was from MedChem Express (New Jersey, United States); dithiothreitol (DTT) and Tris were from BBI Life Sciences (Shanghai, China); rLys-C and trypsin were from Promega Corporation (Madison, United States); triethylammonium bicarbonate (TEAB) buffer, iodoacetamide (IAM) and bovine serum albumin (BSA) were from Sigma-Aldrich Corporation (St Louis, MO, United States); Annexin V-FITC apoptosis detection kit was from Becton, Dickinson and Company (New Jersey, United States); cell cycle analysis kit was from Beyotime Biotechnology (Shanghai, China); acetonitrile (ACN), formic acid (FA) and TMT 6-plex reagent kit were from Thermo Fisher Scientific (Waltham, MA, United States); protease inhibitor cocktail was from Bimake (Houston, Texas, United States).

### 2.2 Cell culture

Huh-7 cells originally taken from a liver tumor patient as a hepatocyte-derived carcinoma cell line were obtained from Cell Bank of Chinese Academy of Sciences (Shanghai, China). The cells were seeded as monolayers in 25-cm^2^ or 75-cm^2^ culture flasks at 4×10^5^ or 1.2×10^6^ cells per flask and cultured in DMEM containing 10% FBS, 100 μg/ml streptomycin, 100 units/ml penicillin and 4.5 g/L glucose. The cell growth was maintained under 5% CO_2_ atmosphere at 37°C until the attached cells reached a confluence of between 80 and 90%, and then passaged at a ratio of 1:2 or 1:3. The third to sixth passages of cells were used in the present study.

### 2.3 Effects of sorafenib, regorafenib and lenvatinib on Huh-7 cell proliferation

**FIGURE 1:**
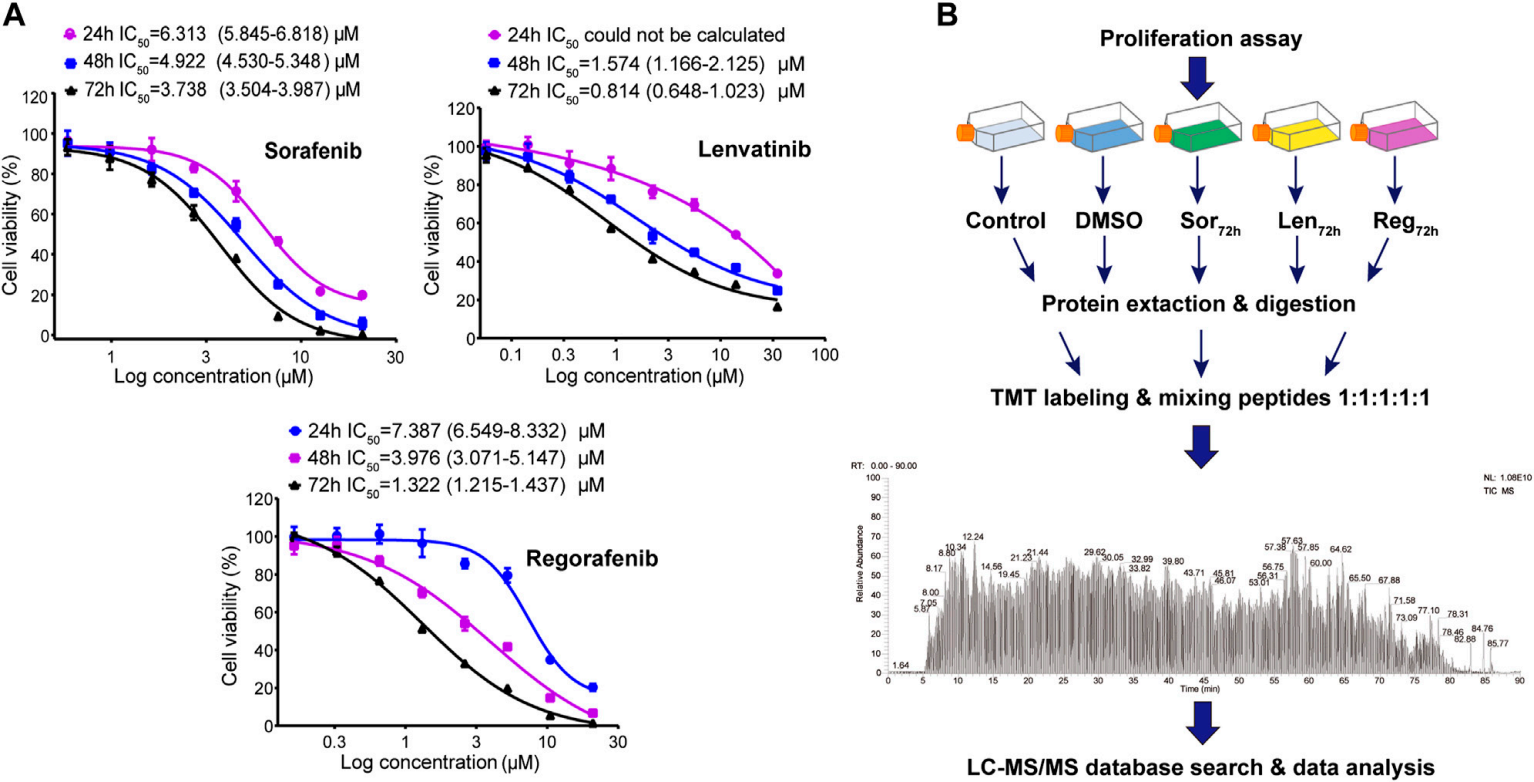
Dose-response curves of the cell growth inhibition by sorafenib-, lenvatinib- and regorafenib-incubation (24, 48 and 72 h) in Huh-7 cells; each point presents mean ± SD, *n* = 6 per concentration **(A)**; and workflow of TMT-based proteomic experiments **(B)**. See Methods for experimental details.

Cell viability was determined by CCK-8 assay. Sorafenib, lenvatinib and regorafenib were respectively dissolved in DMSO at a concentration of 35 mM as stock solutions kept at −20°C until use. The stock solutions were diluted with the culture medium to a series of concentrations of sorafenib (0.59–21 μM), lenvatinib (0.06–35 μM) and regorafenib (0.16–21 μM) immediately before experiments, respectively (Figure 1A). Meanwhile, the blank control (normal saline) medium and solvent control (DMSO) medium were prepared. Cells were seeded in 96-well plates at 5×10^3^ cells per well. Culture medium was changed to the respective sorafenib-, lenvatinib- and regorafenib-containing medium after the cells firmly attached to the surface. Six replicate wells were used for each of the drug concentrations (*n* = 6). After 24, 48 or 72 h of incubation with each inhibitor, we changed the culture medium with fresh medium before adding CCK-8 solution (10:1, *v*/*v*). One hour later, the microplate reader (Infinite 200 Pro, Tecan Austria GmbH, Grodig, Austria) was used to measure the absorbance at 450 nm. Each concentration-response curve of sorafenib, lenvatinib or regorafenib was fit to a nonlinear regression, and the value of IC_50_ was calculated by GraphPad Prism 8.00 software (GraphPad Software Inc, United States).

### 2.4 Cell cycle analysis in Huh-7 cells treated by sorafenib, regorafenib and lenvatinib at IC_50_

We seeded the Huh-7 cells into 75-cm^2^ flasks at a density of 1.2×10^6^ cells/flask and divided them into five experimental groups including normal saline, 0.1‰ DMSO, sorafenib at IC_50_, lenvatinib at IC_50_ and regorafenib at IC_50_ (*n* = 3). When the cells were firmly attached onto the flask bottom, we changed the medium with corresponding drug-containing medium. After 72 h incubation, Huh-7 cell samples (1×10^6^ cells/ml) were prepared by trypsinization and centrifugation (1,000 rpm, 5 min). Following additional washing with cold PBS, we gently resuspended and fixed the cells in 1 ml of 70% ethanol for 24 h at 4°C. According to the instructions of cell cycle analysis kit, each sample was further washed with ice-cold PBS twice, and then we suspended the cells in 535 μL buffer (adding 25 μL propidium iodide (PI) staining solution and 10 μL RNase A into 500 μL buffer), followed by an incubation at 37°C for 30 min in darkness. We used the flow cytometry Coulter Epics XL (Beckman Coulter, United States) to analyze the cell samples, and used the EXPO32-ADC software for the data acquisition and analysis to get the information of cell distribution in different cell cycle phases (G_0_/G_1_, S, and G_2_/M).

### 2.5 Apoptosis analysis in Huh-7 cells treated by sorafenib, regorafenib and lenvatinib at IC_50_

The experimental procedures of cell culture, experimental grouping and cell treatments were the same as the cell cycle assay. After 72 h incubation of Huh-7 cells with normal saline, DMSO, sorafenib, lenvatinib and regorafenib, the cell samples were harvested following trypsinization and centrifugation. According to the instructions of apoptosis detection kit, the cells were washed twice in ice-cold PBS and resuspended to a density of 1×10^6^ cells/ml in 1 ml Binding Buffer. The 100 μL cell suspension was incubated with 5 μL Annexin V-FITC at room temperature for 15 min in the darkness. Thereafter, we added 5 μL PI into the cell suspension for 5 min and 400 μL binding buffer into each cell suspension. The Coulter EPICS XL MCL flow cytometer (Beckman Coulter, United States) was used to analyze the samples, followed by the data acquisition and analysis using the EXPO32-ADC software to get the information of early apoptotic cells (PI negative and Annexin V positive cells), late apoptotic cells (PI and Annexin V positive cells), necrotic cells (PI positive and Annexin V negative cells), and normal cells (PI and Annexin V negative cells).

### 2.6 Proteomics study in Huh-7 cells treated with sorafenib, regorafenib and lenvatinib

#### 2.6.1 Protein sample preparation

The experimental procedures of cell culture, experimental grouping and cell treatments were the same as the cell cycle assay. After 72 h incubation of Huh-7 cells with normal saline, DMSO, sorafenib, lenvatinib and regorafenib, the intact cell samples from each group were washed twice in PBS, and then lysis buffer (8 M urea, 50 mM Tris-HCl, pH 8.0, 1×protease inhibitor cocktail) was added. The cell samples were harvested with the help of a cell scraper, and then lysed with brief sonication (Jy92-INN Ultrasonic Homogenizer, Ningbo Scientz Biotechnology Co., China) to help protein solubilization. After collecting the protein supernatant, protein concentration of each sample was determined by a BCA assay kit.

The 50 μg of extracted protein from each sample was reduced with 20 mM DTT for 1 h followed by an alkylation reaction with 50 mM IAM for 45 min at 37°C in the darkness. Proteins were then pelleted by centrifugation at 8,000 g, 4°C for 10 min after being precipitated by ice-cold acetone overnight. The protein pellets were first digested with Lys-C enzyme (enzyme: substrate = 1:150, *w/w*) at 37°C for 3 h. Trypsin (enzyme: substrate = 1:100, *w/w*) was then added and the digestion process was continued at 37°C overnight. The digestion was finally stopped by adding 10 μL of 10% FA. Peptides were extracted using solid-phase techniques utilizing Sep-Pak tC18 cartridges (Waters, Millford, MA) in accordance with manufacturer’s instructions. The peptides were dried and stored at −80°C.

#### 2.6.2 Tandem mass tag (TMT) labeling and fraction

Peptides were reconstituted in 50 μL TEAB buffer containing 60% CAN. A standard sample for normalization between runs was prepared by combining 10 μg of peptide from each individual sample of five experimental groups. Two TMT 6-plex kits (Thermo Scientific, United States) were used. Each labeling reagents (0.8 mg) was dissolved in 62 μL ACN. Three batches, each batch consisted of five experimental samples and one standard sample, were labeled by different labeling reagents. TMT labeling was performed on 30 μg of peptides from each individual sample and the standard sample.

Each sample containing 30 μg of peptide was combined with 30 μL of its respective 6-plex TMT reagent and incubated for 2 h at 26°C. The labeling reactions were then quenched by adding 5% hydroxylamine for a further 30 min incubation. The samples in each set were mixed in a 1:1:1:1 ratio and dried in a centrifugal evaporator. The mixed TMT-labeled peptides were desalted by the Sep-Pak tC18 cartridges again, and dried in a centrifugal evaporator.

The mixed TMT-labeled peptides were resuspended in 110 μL of 95% buffer A (20 mM NH_3_•H_2_O, 2% ACN, pH 10.5)/5% buffer B (20 mM NH_3_•H_2_O, 98% ACN), and separated utilizing a 1.7 μm × 2.1 mm × 100 mm BEH C18 column (Waters, United States) in a Dionex Ultimate 3000 RSLC system with a 68 min gradient from 5% buffer B to 80% buffer B at a flow rate of 0.1 ml/min. Eluted fractions were pooled into 11 peptide samples over the gradient. Each sample was dried. The peptide samples were reconstituted in water containing 0.1% FA for the following LC-MS/MS analysis.

#### 2.6.3 LC-MS/MS analysis

The TMT-labeled peptides were separated by a Thermo Easy-nLC 1000 HPLC system coupled with an Orbitrap Fusion mass spectrometer using a NanoFlex source (Thermo Scientific, United States). Chromatographic condition and MS method were performed as described previously (Kong et al., 2018). Briefly, the peptides were separated in a home-made C18 column packed in 75 µm ⅹ 20 cm fused silica of Phenomenex chromatographic material (Aqua C18 120 Å, 3 µm) with buffer A (0.1% FA) and buffer B (ACN, 0.1% FA) at gradient elution in 95 min delivered at a flow rate of 300 nL/min. The gradient was as follows: 2–4% B from 0 to 2 min, 4–23% B from 2 to 67 min, 23–60% B from 67 to 80 min, 60–95% B from 80 to 81 min, and 95% B from 81 to 95 min.

For the MS^2^ data acquisition, the data were collected in a data-dependent mode. A full scan in the range of 350–1,550 m/z with a resolution of 60,000 at m/z 200 was acquired for the MS^1^. The quadrupole was used to isolate MS^2^ ions with an isolation window of 1.6 m/z. HCD (high energy collisional dissociation) fragmentation with 38% normalized collision energy was used for ion fragmentation. Fragment ions were analyzed with a resolution of 15,000 at m/z 200. The duty cycle was set to 3 s. For MS^1^ and MS^2^ scans, the automatic gain control (AGC) settings were 2 × e^5^ and 5 × e^4^ ions, with maximum ion injection times of 50 and 100 ms, respectively.

#### 2.6.4 Protein identification, quantification and bioinformatic analysis

Raw data were analyzed against the Homo species proteome database using SEQUEST HT in Proteome Discoverer 2.1 software (Thermo Scientific, United States). The default settings were used with an exception of allowing for two missed cleavages; a parent ion tolerance of 10 ppm and a fragment mass tolerance of 0.02 Da were set; and a false discovery rate (FDR) cutoff value of 1% was used at the peptide and protein levels. As a fixed modification, carbamidomethyl (+57.021 Da) was chosen. As variable modifications, TMT reagents (+229.163 Da) on lysines, oxidation of methionine residues (+15.9949 Da), and N-terminal acetylation (+42.011 Da) were used.

Two normalization procedures were used to handle the 20-plex experiment (5 TMT experiments with four channels each) according to the reference (Plubell et al., 2017). The first normalization was performed within each TMT experiment. To adjust the total intensity to the average total intensity across the four channels, the grand total reporter ion intensity for each channel was multiplied by global scaling factors. Then, normalization for each protein was performed according to the average values in the common pool channels within each TMT experiment. Differential protein abundance between groups was determined by comparing the normalized total reporter ion intensities.

The PCA analysis, HCA analysis, Venn diagram and volcano plot were conducted in 'Wu Kong’ platform (https://www.omicsolution.com/wkomics/main/). The differential proteins for the comparison of two groups were defined with *p* value ≤0.05, and fold-change ≥ 1.20 or ≤0.83. Functional enrichment analysis of the differential proteins, including KEGG (Kyoto Encyclopedia of Genes and Genomes) pathway analysis was carried out on the DAVID online platform (2021 Update, https://david-d.ncifcrf.gov/tools. jsp).

### 2.7 Other statistical analysis

All data were represented as mean ± standard deviation (mean ± SD). The IC_50_ values were calculated via nonlinear regression model and expressed as means with 95% confidence interval. In the experiments of cell proliferation, cell cycle, cell apoptosis and downregulated protein analysis, significant differences were determined using a one-way analysis of variance (ANOVA) followed by Bonferroni’s multiple comparison test using GraphPad Prism 8.0 (GraphPad Software, San Diego, CA, United States) and *p* value <0.05 was considered statistically significant.

## 3 Results and discussion

### 3.1 Effects of sorafenib, regorafenib and lenvatinib on Huh-7 cell proliferation

Since lenvatinib at ∼10 µM does not affect the proliferation of HepG2 cells (Sasaki et al., 2020) and its plasma concentration in Japanese cancer patients is 0.1 µM (Nagahama et al., 2019), the present study established the satisfactory dose-response curves of the cell growth inhibition by sorafenib, regorafenib and lenvatinib in Huh-7 cells. Sorafenib, regorafenib and lenvatinib inhibited the proliferation of Huh-7 cells in a concentration- or time-dependent manner, respectively (Figure 1A). The IC_50_ values following 24, 48 and 72 h of incubation with Huh-7 cells were respectively 6.313 (5.845–6.818) µM, 4.922 (4.530–5.348) µM and 3.738 (3.504–3.987) µM for sorafenib; and 7.387 (6.549–8.332) µM, 3.976 (3.071–5.147) µM and 1.322 (1.215–1.437) µM for regorafenib. The IC_50_ values of lenvatinib following 48 and 72 h of incubation with Huh-7 cells were 1.574 (1.166–2.125) µM and 0.814 (0.648–1.023) µM, but the IC_50_ of lenvatinib after 24 h of incubation was not able to be calculated. Recently, we reported that IC_50_ value of sorafenib in Huh-7 cells after 72 h incubation was 3.5 (3.39–3.62) µM (Bai et al., 2019), which is the same as that obtained in the present study. Therefore, the incubation time of Huh-7 cells with the three multi-RTK inhibitors was determined to be 72 h in the following experiments.

After 72 h incubation of Huh-7 cells with the three inhibitors, the IC_50_ value of lenvatinib was significantly smaller than that of regorafenib (0.814 versus 1.322 µM; *p* < 0.01, one-way ANOVA followed by Bonferroni’s multiple comparison test), and regorafenib IC_50_ was significantly smaller than sorafenib (1.322 versus 3.738 µM; *p* < 0.01). Maximal inhibition rate of the Huh-7 cell proliferation by 72 h incubation with sorafenib (21 µM), regorafenib (21 µM) or lenvatinib (35 µM) reached up to 99.8 ± 0.2 (%), 98.7 ± 0.1 (%) or 83.4 ± 0.5 (%) (Figure 1A). Though the inhibition potency against cell proliferation by lenvatinib was stronger than that by sorafenib or regorafenib, its maximal inhibition was weaker than sorafenib and regorafenib. Sorafenib is the most recommended first-line treatment for advanced-stage HCC patients, but the SHARP trial showed an improvement of the median overall survival duration from 7.9 to 10.7 months (Llovet et al., 2008). In recent years, lenvatinib and a second-line agent of regorafenib have been approved and improved the clinical outcomes, but the median overall survival remains ∼1 year in most patients with advanced HCC (Bruix et al., 2017; Kudo et al., 2018). Thus, even in patients with HCC initially responded to the treatment of multi-RTK inhibitors, a drug-resistance invariably develops, and it is well-documented for sorafenib that compensatory signaling pathways of HCC would be activated (Firtina Karagonlar et al., 2016; Abou-Alfa et al., 2018). Therefore, there is a pressing need for novel therapeutic strategies to extend the lives of patients with advanced HCC.

### 3.2 Effects of sorafenib, regorafenib and lenvatinib on cell cycle and apoptosis of Huh-7 cells

**FIGURE 2:**
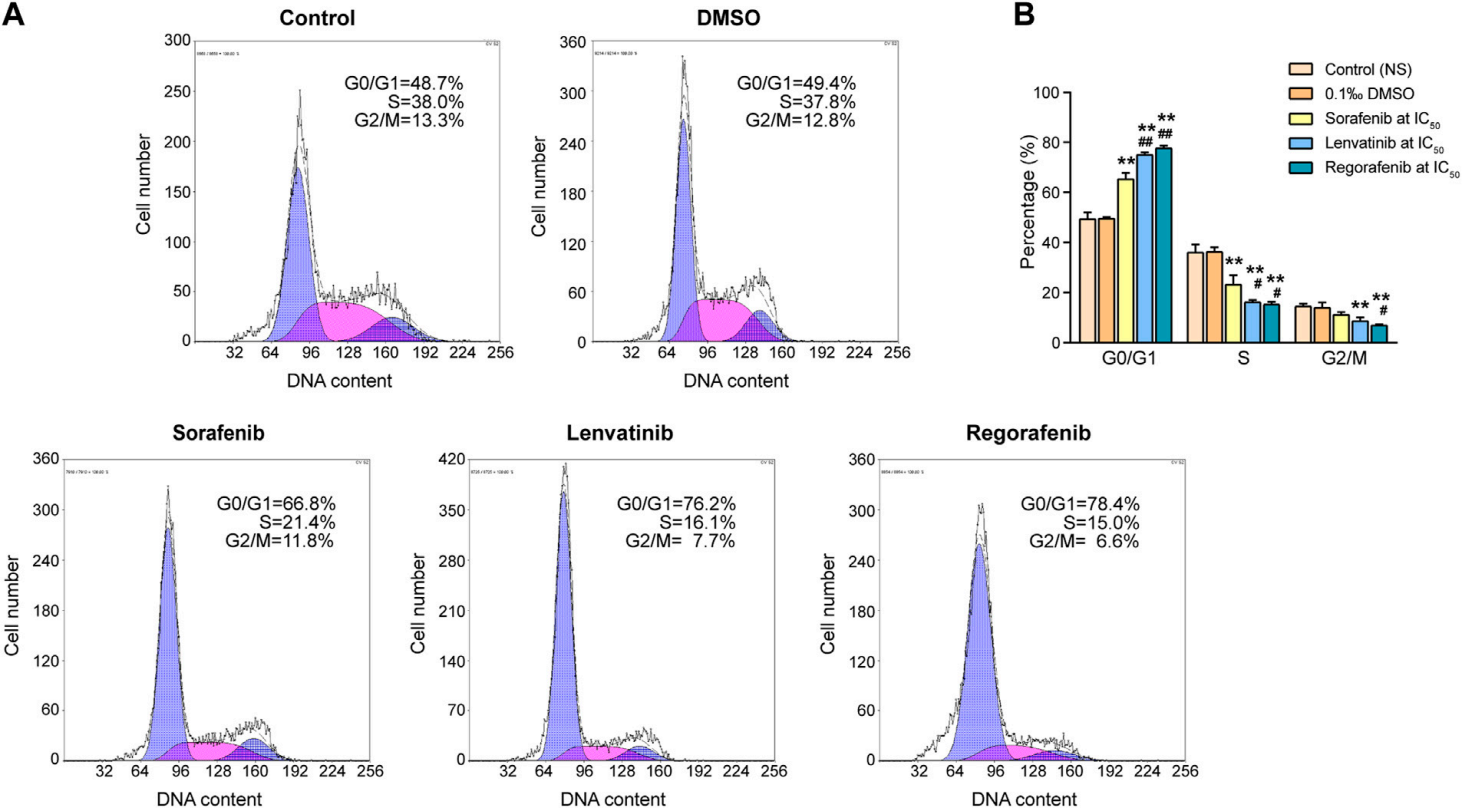
Effects of 72 h incubation with respective sorafenib, lenvatinib and regorafenib at their IC_50_ on cell cycle progression in Huh-7 cells **(A)** Representative histograms from sorting of propidium iodide-stained cells by flow cytometry **(B)** Bar graph showing the percentage of cells in various phases of cell cycle distribution. Each bar presents mean ± SD (*n* = 3). The **p < 0.01 versus DMSO; ^#^ *p* < 0.05 and ^##^ *p* < 0.01 versus sorafenib. NS, normal saline.

Sasaki et al. (2020) indicated that one of the key requirements for cellular growth and proliferation is the fast cell cycle progression, so that the cellular proliferation inhibition is often accompanied by cell cycle arrest (Sasaki et al., 2020). As shown in Figure 2, DMSO at final concentration of 0.1‰ did not induce any significant effects on cell cycle of Huh-7 cells when compared with the control, but 72 h of incubations with sorafenib, lenvatinib and regorafenib at the respective IC_50_ exhibited different effects in different cell cycle phases. Figure 2B shows a significantly higher percentage of cells in G_0_/G_1_ phase (65.4%) and a significantly lower percentage of cells in S phase (23.3%) in sorafenib group, comparing to the G_0_/G_1_ phase (49.6%) and S phase (36.3%) in DMSO group (*p* < 0.01, one-way ANOVA followed by Bonferroni’s multiple comparison test). Lenvatinib and regorafenib had similar effects to sorafenib on G_0_/G_1_ phase cells and S phase cells, but their effects were significantly stronger than sorafenib, respectively (*p* < 0.05 and *p* < 0.01). Percentages of cells in G_2_/M phase of DMSO, sorafenib, lenvatinib and regorafenib groups were 14.1%, 11.2%, 8.7% and 7.0%, respectively. In comparison with DMSO group, either lenvatinib or regorafenib significantly deceased the percentage of cells in G_2_/M phase (*p* < 0.01), but the inhibitory effect of sorafenib was not significant (*p* > 0.05, Figure 2B). Meanwhile, the inhibitory effect on G_2_/M phase of regorafenib was significantly stronger than that of sorafenib (*p* < 0.05).

**FIGURE 3:**
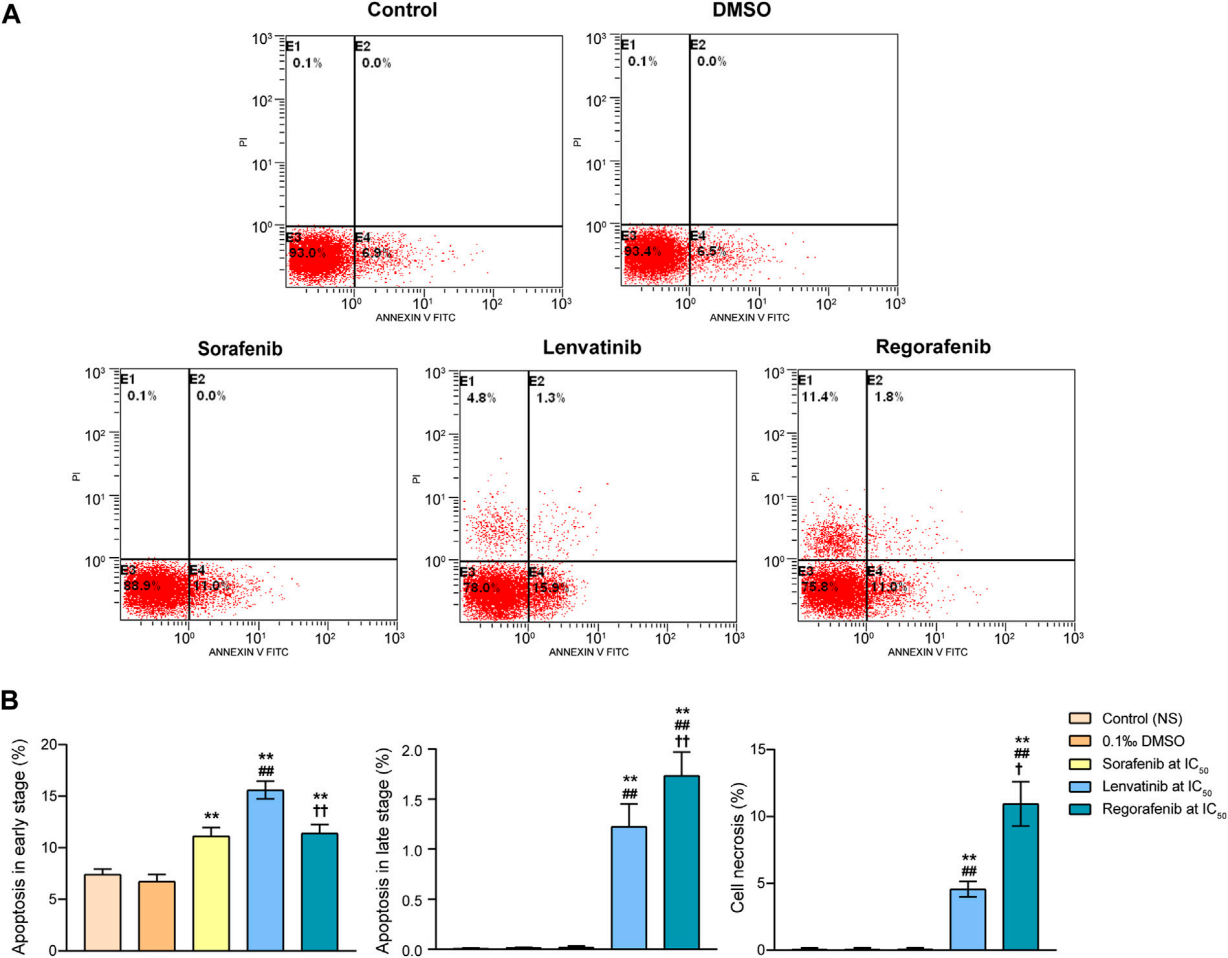
Effects of 72 h incubation with respective sorafenib, lenvatinib and regorafenib at their IC_50_ on cell apoptosis in Huh-7 cells **(A)** Representative histograms from sorting of Annexin V/propidium iodide double-stained cells by flow cytometry. Cells in quadrants of E1, E2, E3 and E4 present necrotic, late apoptotic, viable and early apoptotic populations, respectively **(B)** Bar graphs showing the percentage of cells in early apoptotic, late apoptotic and necrotic populations. Each bar presents mean ± SD (*n* = 3). The **p < 0.01 versus DMSO; ^##^ *p* < 0.01 versus sorafenib; ^†^ *p* < 0.05 and ^††^ *p* < 0.01 versus lenvatinib. NS, normal saline.

To determine whether the proliferation inhibition of Huh-7 cells by the three inhibitors was related to their induction of cell apoptosis, we further quantified the apoptotic populations in the Huh-7 cells using flow cytometry with Annexin V/propidium iodide staining. Cells can be distinguished into necrotic, late apoptotic, viable and early apoptotic populations in quadrants of E1, E2, E3 and E4, respectively (Figure 3A). DMSO did not induce any significant changes of Huh-7 cell populations in the four quadrants when compared with the control (*p* > 0.05). Sorafenib at IC_50_ significantly increased only the proportion of early apoptotic cells from 6.7% of DMSO group to 11.1% (*p* < 0.01, Figure 3B). Moreover, the early apoptotic cells, late apoptotic cells, and necrotic cells in lenvatinib group significantly were increased by 1.3, 121 and 64 times when compared with DMSO group, respectively (*p* < 0.01). Especially, regorafenib at IC_50_ significantly increased the late apoptotic cells and necrotic cells by 0.4 and 1.4 times when compared with lenvatinib group (*p* < 0.05 and *p* < 0.01, Figure 3B).

These findings obtained from both experiments of cell cycle and apoptosis suggested that while sorafenib, lenvatinib and regorafenib all drove the remaining surviving Huh-7 cells into a G_0_/G_1_ arrest, accompanied by a decrease in the number of S phase cells, lenvatinib and regorafenib had more effective induction of cell cycle arrest at G_0_/G_1_ phase than sorafenib. Notably, lenvatinib produced a much stronger induction of Huh-7 cells into early apoptosis than sorafenib and regorafenib, but the proportion of necrotic cells induced by regorafenib was 2.4 times as large as that induced by lenvatinib. The present experimental observations provided new evidences supporting the current state of the clinical use of the three multi-RTK inhibitors, i.e., sorafenib reported by Bouattour et al. (2019) is still the first-line standard of care for many patients with advanced HCC, including patients with locally advanced HCC and HCV-related advanced HCC (Bouattour et al., 2019); lenvatinib reported by Kudo et al. (2018) may have more benefit than sorafenib in HBV infected patients based on a subgroup analysis of the phase III clinical trials (Kudo et al., 2018); and regorafenib has been approved by the FDA and EMA as the second-line treatment after sorafenib failure in patients with advanced HCC (Bouattour et al., 2019).

### 3.3 Proteomic analysis of Huh-7 cells treated by sorafenib, regorafenib and lenvatinib

#### 3.3.1 Proteomic data analysis by multivariate statistical methods

To analyze the relationships between proteomic changes and the different effects on cell cycle and apoptosis induced by the three inhibitors in Huh-7 cells, TMT-based quantitative proteomics technique was used to measure the protein expression levels of 15 samples collected from five experimental groups including normal saline, 0.1‰ DMSO, IC_50_ sorafenib, IC_50_ lenvatinib and IC_50_ regorafenib (*n* = 3). Figure 1B is the general scheme of the present proteomic study. The 11,577 proteins in Huh-7 cells were identified and quantified using the TMT-labeled proteomic method, and the Supplementary Figure S1A shows a representative MS^2^ spectrum of the peptides indicating how the peptide sequences were identified by the Proteome Discoverer software (Version 2.1, Thermo Corporation, CA, United States). The Supplementary Figure S1B-D indicates maximal missed cleavage sites, TMT labeling efficiency and distribution of the special peptides.

**FIGURE 4:**
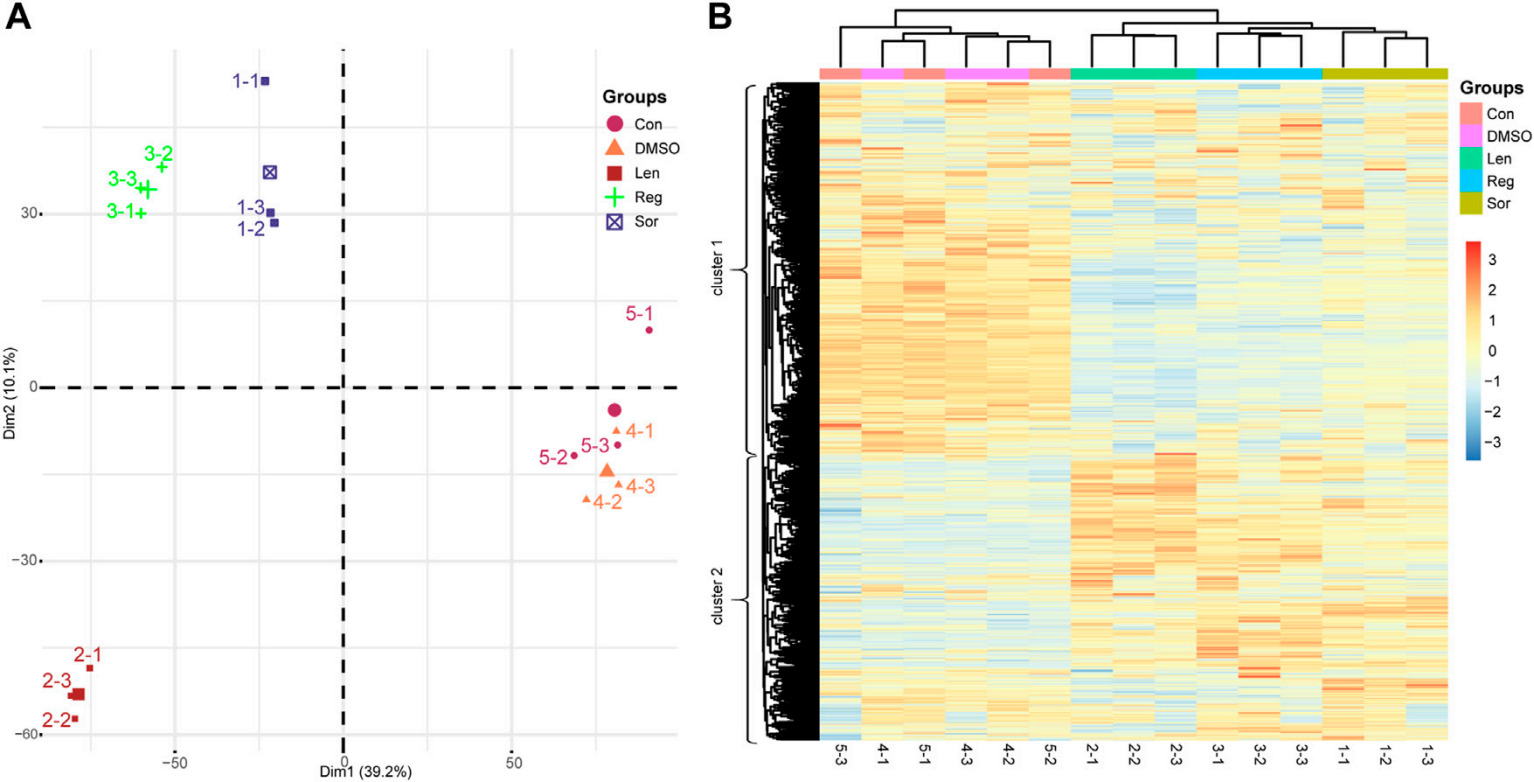
Principal component analysis **(A)** and hierarchical clustering analysis **(B)** based on the proteomic data obtained from the Huh-7 cells treated by 72 h incubation with respective sorafenib (Sor), lenvatinib (Len) and regorafenib (Reg) at their IC_50_ as well as normal saline (Con) and 0.1‰ DMSO. The five experimental groups are indicated by different colors and numbers (1–5). For heatmap, samples are shown as columns; and proteins arrayed in rows; branch length indicates the degree of variance; and color in row presents the normalized regulated level of the proteins. Each group contains three samples (*n* = 3).

Principal component analysis (PCA) is one of the most widely used multivariate analysis technique to reduce dimensionality in multivariate data and is useful to identify the grouping of samples in the whole detected proteins. To better understand the influence of DMSO and the effects of sorafenib, lenvatinib and regorafenib at the respective IC_50_ on the protein turnover of Huh-7 cells, we tried to interpret the identified and quantified 11,577 proteins using PCA. As shown in Supplementary Figure S2, PC1 accounts for the most variant components (39.2%) and PC2 represents the other additional 10.1% among all the variant components. The PCA score plot (Figure 4A) with PC1 and PC2 revealed clear differences in the identified proteins among sorafenib, lenvatinib and regorafenib. Firstly, the samples in sorafenib, lenvatinib and regorafenib groups were significantly separated from those in control and DMSO groups in the PC1 direction (horizontal axis), but the samples in both DMSO and control groups were overlapped almost completely. Secondly, the groups of sorafenib, lenvatinib and regorafenib were clearly separated from each other, especially the samples in lenvatinib group were separated far away from those in sorafenib and regorafenib groups in the PC2 direction (vertical axis) (Figure 4A). As shown in Supplementary Figure S3, the three multi-RTK inhibitors belong to a class of phenylurea derivatives as antitumor agents. The only difference between sorafenib and regorafenib is that regorafenib has a fluorine atom in the central benzene ring. However, the chemical structure of lenvatinib is quite different from that of sorafenib and regorafenib.

In the left dendrogram of Figure 4B, an HCA analysis, the horizontal direction represents the distance or dissimilarity between proteins or clusters. Each sample (columns of Figure 4B) in the cluster 1 and cluster 2 contained 6,521 proteins and 5,056 proteins, respectively. In general, most of the proteins in cluster 1 obtained from sorafenib, lenvatinib and regorafenib groups were obviously downregulated (Figure 4B). On the contrary, most of the proteins in cluster 2 obtained from sorafenib, lenvatinib and regorafenib groups were obviously upregulated (Figure 4B). Like PCA results, the samples in sorafenib, lenvatinib or regorafenib group were clustered together within each individual group, and all of them were clearly separated from those in DMSO and control groups. However, the effects and magnitude of the effects induced by regorafenib on cell cycle and cell apoptosis in Huh-7 cells were much more like that induced by lenvatinib but not sorafenib (Figures 2, 3). Recently, Bai et al. (2019) reported that 520 unique proteins were significantly upregulated by sorafenib in HepG2 cells surviving from the attack of high concentration sorafenib, and bioinformatics-assisted analysis of those proteins revealed that the metabolic pathways were mainly involved (Bai et al., 2019). Therefore, it was necessary to further analyze the differential proteins regulated commonly by the three inhibitors to understand their respective characteristics in the regulation of signaling pathways responsible for the cell cycle arrest and apoptosis induction.

#### 3.3.2 Identified proteins regulated commonly by the three inhibitors

**FIGURE 5:**
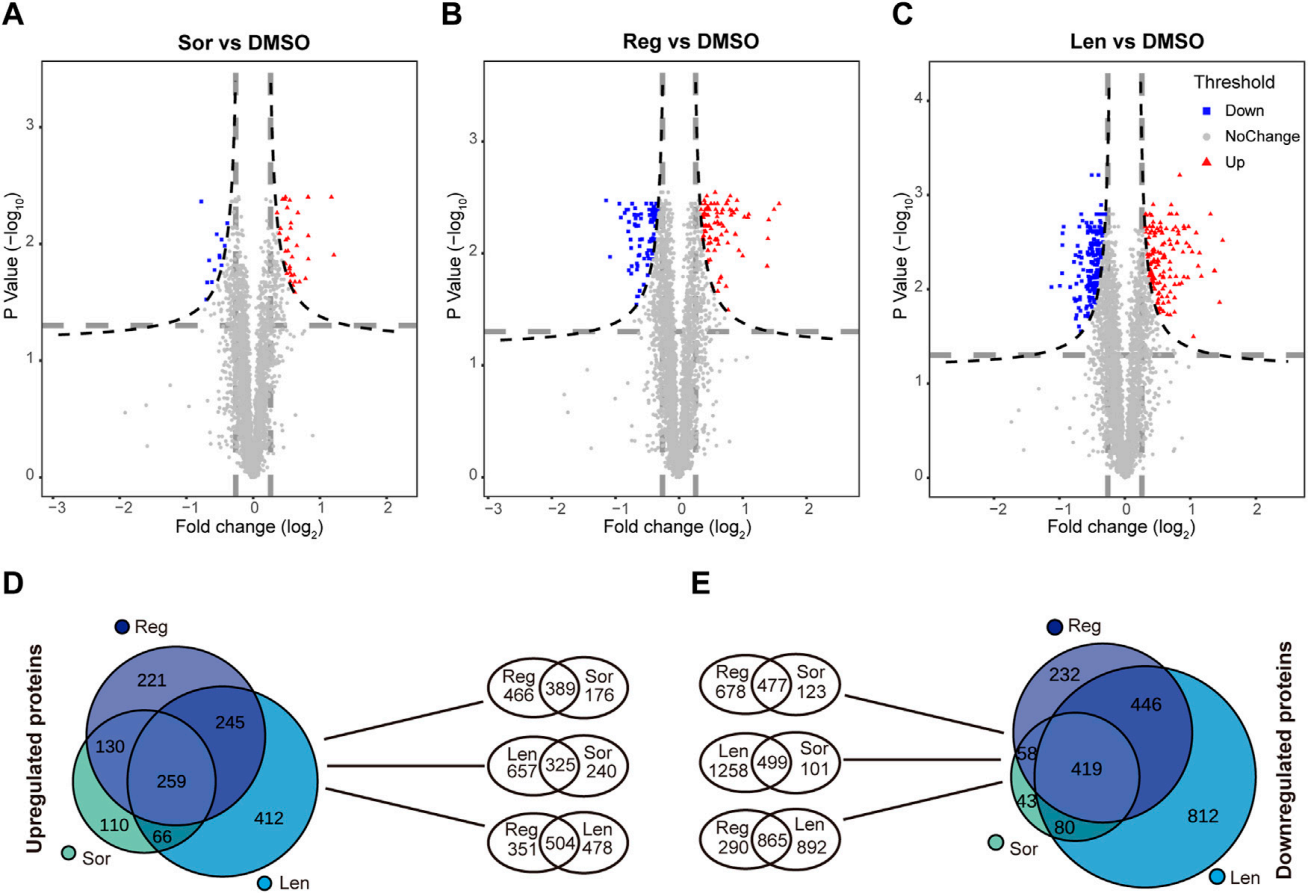
Volcano plots of significantly altered proteins in Huh-7 cells treated by 72 h incubation with respective sorafenib (Sor), **(A)**, lenvatinib (Len), **(B)** and regorafenib (Reg), **(C)** at their IC_50_ when compared with 0.1‰ DMSO. Venn diagrams show the comparison among the upregulated proteins **(D)** and the downregulated proteins **(E)** in Huh-7 cells treated by Sor, Len and Reg at their IC_50_.

Volcano plots (Figure 5A–C) were designed to show the relationship between fold change in proteins (significant upregulation in red color and significant downregulation in blue color) and the level of significance (*p* < 0.05). Sorafenib, lenvatinib and regorafenib at IC_50_ significantly and respectively upregulated 565, 982 and 855 proteins, and downregulated 600, 1,757 and 1,155 proteins when compared with DMSO group (Figures 5D,E). Meanwhile, we found 259 upregulated and 419 downregulated (Supplementary Data) proteins which were commonly regulated by the three multi-RTK inhibitors.

The upregulated 259 proteins were analyzed by David on-line platform, and 15 KEGG pathways significantly enriched (*p* < 0.05) were biosynthesis of antibiotics; fatty acid degradation; valine, leucine and isoleucine degradation; pyruvate metabolism; metabolic pathways; glycolysis/gluconeogenesis; carbon metabolism; etc. (Table 1). The results of KEGG pathway analysis for the upregulated 259 proteins were very similar to our previous investigation of sorafenib in HepG2 cells (Bai et al., 2019).

**TABLE 1:** KEGG pathways enriched by the commonly upregulated proteins in Huh7 cells incubated with sorafenib, lenvatinib and regorafenib at the respective IC_50_ for 72 h.

Name	Count	*p* Value	Fold enrichment
Biosynthesis of antibiotics	14	3.43 × 10^–08^	7.211
Metabolic pathways	30	1.17 × 10^–07^	2.687
Glycine, serine and threonine metabolism	6	2.30 × 10^–05^	16.799
Fatty acid degradation	6	3.32 × 10^–05^	15.599
Histidine metabolism	5	3.88 × 10^–05^	24.816
Valine, leucine and isoleucine degradation	6	5.79 × 10^–05^	13.939
Arginine and proline metabolism	6	7.83 × 10^–05^	13.103
Glycolysis/Gluconeogenesis	6	3.18 × 10^–04^	9.778
Tryptophan metabolism	5	4.29 × 10^–04^	13.649
beta-Alanine metabolism	4	2.62 × 10^–03^	14.089
Pyruvate metabolism	4	5.43 × 10^–03^	10.919
Carbon metabolism	5	1.86 × 10^–02^	4.831
Ascorbate and aldarate metabolism	3	2.42 × 10^–02^	12.132
Metabolism of xenobiotics by cytochrome P450	4	2.87 × 10^–02^	5.902
Tyrosine metabolism	3	3.93 × 10^–02^	9.359

### 3.4 Signaling pathways involved in the proteins downregulated commonly by the three inhibitors

Obviously, the number (419 proteins) of commonly downregulated proteins by the three inhibitors was much more than that of commonly upregulated proteins. Then we tried to analyze the 419 proteins, and 10 KEGG pathways were significantly enriched (*p* < 0.05). The ranking of the top six KEGG pathways were spliceosome; DNA replication; cell cycle; mRNA surveillance; P53 and nucleotide excision repair pathways. Since the six KEGG pathways had a direct relationship to the effects of the three inhibitors on the Huh-7 cell apoptosis and cell cycle, we elucidated the biological meaning of each identified protein basing on the individual KEGG pathway.

#### 3.4.1 Cell cycle signaling pathway

**FIGURE 6:**
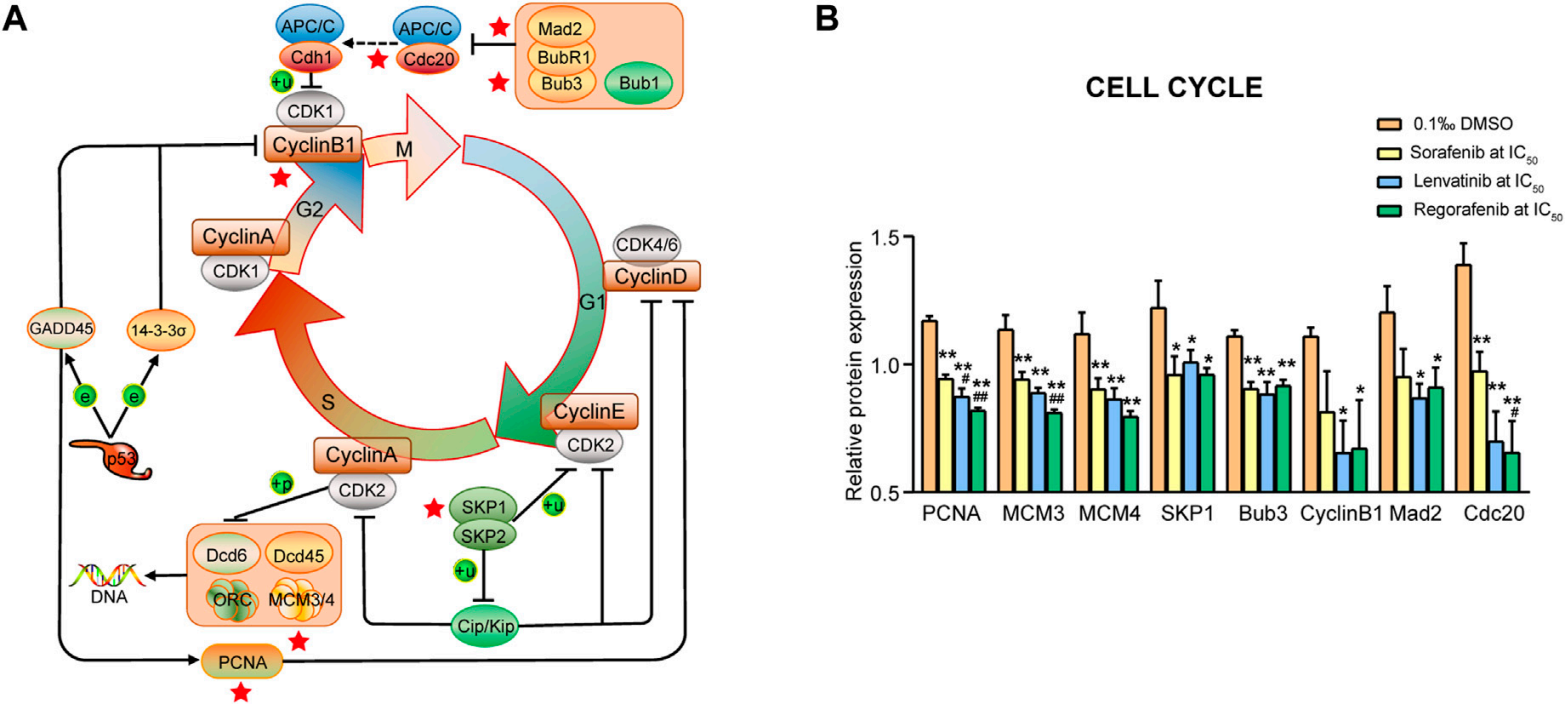
Commonly downregulated proteins involved in the cell cycle signaling pathway **(A)** and their relative expression **(B)** in Huh-7 cells treated by 72 h incubation with respective sorafenib, lenvatinib and regorafenib. Each bar presents mean ± SD (*n* = 3). The *p < 0.05 and **p < 0.01 versus DMSO; ^#^ *p* < 0.05 and ^##^ *p* < 0.01 versus sorafenib. Star symbol in red color indicating the downregulation of the protein.

The cell cycle is the series of molecular events that allow cells to undergo a process of duplicating itself, including five principal phases of G_0_, G_1_, S, G_2_ and M. In the present study, we found eight proteins involved in the cell cycle signaling pathway. Proteins produced by the cells in G_1_ phase are essential for S phase, primarily the proteins relevant to chromosomal replication. The PCNA protein was related to the processes (Figure 6A) and significantly downregulated by sorafenib, lenvatinib and regorafenib, respectively (*p* < 0.01, one-way ANOVA followed by Bonferroni’s multiple comparison test, Figure 6B). Moreover, lenvatinib and regorafenib downregulated PCNA expression more strongly than sorafenib (*p* < 0.05 and *p* < 0.01). PCNA expression was reported to be increased from the late G_1_ phase through the S phase, and it is a marker of cell proliferation, especially in tumors. PCNA can be used as a biomarker for colorecta cancer (CRC) proliferation (Yang et al., 1996).

The cells in S phase duplicate their chromosomes and synthesize histones. Four proteins (MCM3, MCM4, SKP1 and Bub3; Figure 6A) were involved in the processes and significantly downregulated by sorafenib, lenvatinib and regorafenib, respectively (*p* < 0.05 and *p* < 0.01, Figure 6B). Regorafenib downregulated MCM3 expression more strongly than sorafenib (*p* < 0.01). Especially, regorafenib downregulated MCM3 and MCM4 expressions by at least 28% when compared with DMSO (Figure 6B). MCM2, MCM3, MCM4, MCM5, MCM6 and MCM7 are named as MCM2-7 (Figure 10A), which are involved in the traveling replication forks and DNA elongation process. Labib et al. (2000) indicated that the inactivation, disruption, or absence of any part of MCM2-7 perturbs replisome progression (Labib et al., 2000). Meanwhile, an upregulation of MCM4 in liver cancer tissues was reported to be negatively related to the survival of HCC patients (Zheng et al., 2021). Specific silencing of SKP1 gene expression induces the increase in replication stress including DNA double strand breaks and chromothripsis events (Thompson et al., 2020). SKP1 overexpression is promotable to the stemness of CRC cells and predicts poor prognosis of CRC patients (Tian et al., 2020). Bub3 is needed for kinetochore recruitment of spindle checkpoint proteins BubR1 and Bub1. Bub3 is upregulated in oral squamous cell carcinoma cells, and the inhibition of Bub3 can enhance the chemosensitivity to cisplatin (Silva et al., 2019).

Concerning G_2_/M phases, three proteins (Cyclin B1, Mad2 and Cdc20) were involved (Figure 6A). Lenvatinib and regorafenib, but not sorafenib, significantly downregulated Cyclin B1 and Mad2, respectively (*p* < 0.05, Figure 6B). The three inhibitors significantly downregulated the Cdc20 expression, while the effect of regorafenib was stronger than that of sorafenib (*p* < 0.05). Of particular note, the Cdc20 expression levels in the lenvatinib and regorafenib groups decreased to 50.4 and 46.8% of that in the DMSO group, and to 72.2 and 67% of that in sorafenib group (Figure 6B). Like the Cdc20 expression, similar results were observed in Cyclin B1 expression (to 59 and 60.6% of DMSO group, and to 80.4% and 82.5% of sorafenib group, Figure 6B). Brown et al. (2008) reported that estrogen and progesterone lower Cyclin B1 expression, and trigger apoptosis in human adrenal carcinoma cell cultures (Brown et al., 2008). Increasing evidence indicates that the Cyclin B1 suppression could be an essential tool for exerting antiproliferative and proapoptotic action in future antitumor therapy (Yuan et al., 2004). Mad2 acts cooperatively with BubR1 for prevention of premature separation of sister chromatids. Mad2 overexpression was reported to play an important role in the progression of HCC (Zhang et al., 2008). Moreover, Li et al. (2014) reported that Cdc20 is required for full activation of APC/C in M phase in regulating timely cell cycle progression, and an increased Cdc20 expression is related to the progression of HCC (Li et al., 2014).

#### 3.4.2 P53 signaling pathway

**FIGURE 7:**
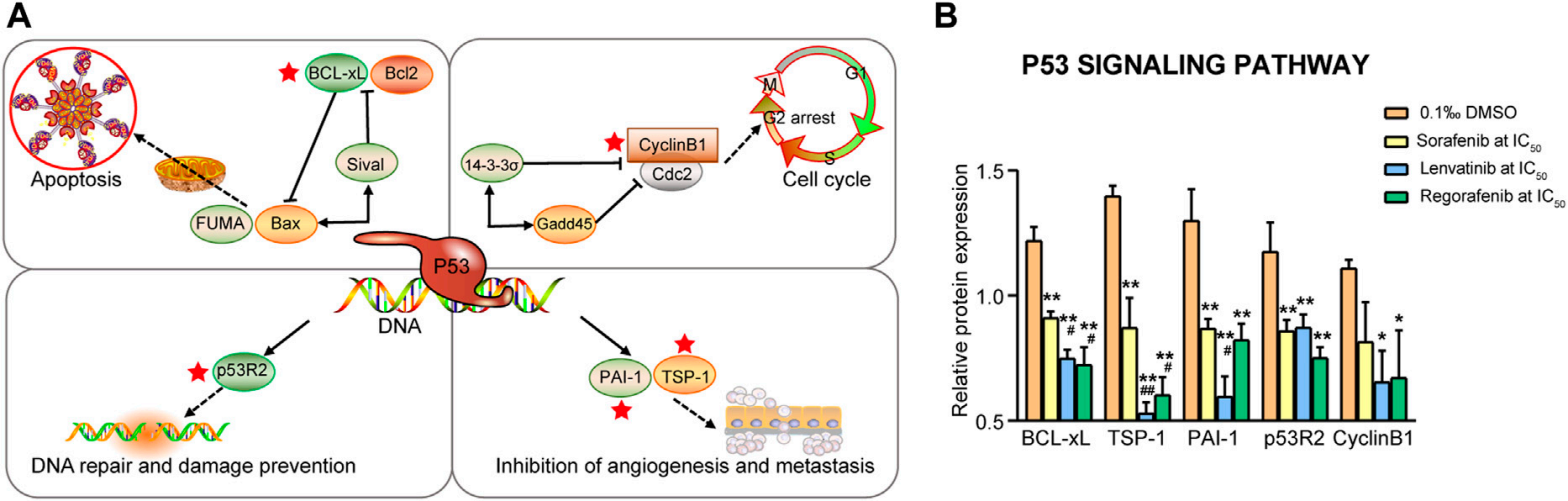
Commonly downregulated proteins involved in the p53 signaling pathway **(A)** and their relative expression **(B)** in Huh-7 cells treated by 72 h incubation with respective sorafenib, lenvatinib and regorafenib. Each bar presents mean ± SD (*n* = 3). The *p < 0.05 and **p < 0.01 versus DMSO; ^#^ *p* < 0.05 and ^##^ *p* < 0.01 versus sorafenib. Star symbol in red color indicating the downregulation of the protein.

It is well-known that p53 is able to regulate the Bcl-2 to Bax expression ratio to control the mitochondrial pathway of cell apoptosis. Five proteins (BCL-xL, TSP-1, PAI-1, p53R2 and Cyclin B1) were related to these processes (Figure 7A), and all the proteins, except for Cyclin B1, were significantly downregulated by sorafenib, lenvatinib and regorafenib, respectively (*p* < 0.01, Figure 7B). Moreover, lenvatinib and regorafenib downregulated the expression of BCL-xL and TSP-1 more strongly than sorafenib (*p* < 0.05 and *p* < 0.01). It is noteworthy that the TSP-1 and PAI-1 expression levels in the lenvatinib group decreased to 38% and 45.9% of that in the DMSO group, and to 60.9% and 68.7% of that in sorafenib group (*p* < 0.05, Figure 7B). Follis et al. (2013) reported that the PUMA-induced partial unfolding of BCL-xL disrupted the interaction between cytosolic p53 and BCL-xL, releasing the bound p53 to trigger the cell apoptosis (Follis et al., 2013). BCL-xL is the sole protein strongly upregulated in human CRC specimens among the anti-apoptotic Bcl-2 proteins (Scherr et al., 2016). An upregulation of active Caspase-3 was found to be consistently associated with the TSP-1-mediated apoptosis (Li et al., 2003). Rath et al. (2006) reported that induction of apoptosis by anticancer agents of camptothecin or doxorubicin in human thyroid cancer cells was greatly dependent on the downregulation of TSP-1 expression (Rath et al., 2006). PAI-1 level was reported to be increased in many solid tumors, and the increase is consistently associated with shorter length of patient survival. Since PAI-1 may play a significant role in regulating cancer cell apoptosis, inhibitors of PAI-1 might be useful as an anti-cancer therapy (Lademann et al., 2005). Yamaguchi et al. (2001) have reported that p53R2-dependent DNA synthesis plays a pivotal role in cell survival by repairing damaged DNA in the nucleus, and dysfunction of this pathway might result in activation of p53-dependent apoptosis (Yamaguchi et al., 2001). Okumura et al. (2006) have demonstrated that a positive p53R2 expression is significantly correlated with depth of invasion, lymph node metastasis and poor prognosis in patients with esophageal squamous cell carcinoma (Okumura et al., 2006). The relationship between Cyclin B1 and cell apoptosis has been described above.

#### 3.4.3 Spliceosome signaling pathway

**FIGURE 8:**
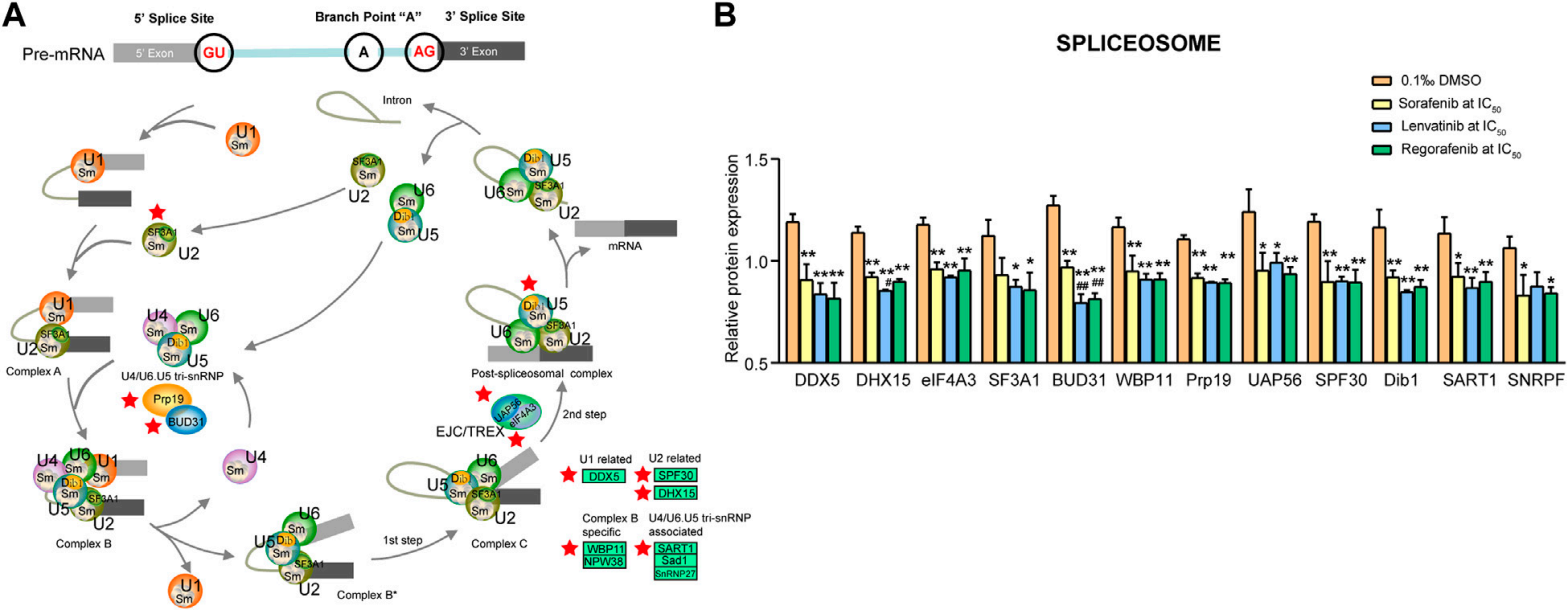
Commonly downregulated proteins involved in the spliceosome pathway **(A)** and their relative expression **(B)** in Huh-7 cells treated by 72 h incubation with respective sorafenib, lenvatinib and regorafenib. Each bar presents mean ± SD (*n* = 3). The *p < 0.05 and **p < 0.01 versus DMSO; ^#^ *p* < 0.05 and ^##^ *p* < 0.01 versus sorafenib. Star symbol in red color indicating the downregulation of the protein.

Vinson (2015) indicated that the enzyme of spliceosome removes introns from the transcribed pre-mRNA, and it comprises of more than 100 associated proteins and five small nuclear ribonucleoproteins including U1, U2, U4, U5 and U6 (Vinson, 2015). In the present study, we found 12 downregulated proteins involved in the spliceosome signaling pathway (Figure 8A).

Shen and Pelletier (2020) reported that DExD/H-box RNA helicase genes including 37 DDX (DEAD-box) and 17 DHX (DEAH-box) genes play myriad roles in processes ranging from transcription and mRNA-protein complex remodeling to RNA decay and translation (Shen and Pelletier, 2020). Three proteins (DDX5, DHX15 and eIF4A3) were related to these processes and significantly downregulated by sorafenib, lenvatinib and regorafenib, respectively (*p* < 0.01, Figure 8B). Lenvatinib downregulated DHX15 expression more strongly than sorafenib (*p* < 0.05). DDX5 protein has key roles in U1 snRNP-5′ splice site complex stability and spliceosome assembly, which is overexpressed in colorectal tumors, suggesting an important role of DDX5 in tumor development (Clark et al., 2008). The recruitment of U2 snRNP to the branch point sequence of an intron is a critical step of pre-mRNA splicing, and DHX15 provides a quality control in U2 snRNP-related engagement with an intron (Maul-Newby et al., 2022). Upregulation of DDX5 promoted proliferation of non-small cell lung cancer (NSCLC) cells *in vivo* and *in vitro*, whereas the downregulated DDX5 produced the opposite effects (Wang et al., 2015). eIF4A3, one of DDX family members, is found in the nucleus, which functions to promote unwinding of secondary structures within pre-mRNA to stimulate the splicing reaction (Chan et al., 2004). Circ_cse1L can downregulate the expression of PCNA by binding to eIF4A3, thereby inhibiting the proliferation of CRC cells (Xu et al., 2020).

Concerning pre-mRNA splicing factors, five proteins (WBP11, BUD31, Prp19, SF3A1 and UAP56) were involved (Figure 8A). All of them, except for SF3A1, were significantly downregulated by sorafenib, lenvatinib and regorafenib, respectively (*p* < 0.05 and *p* < 0.01, Figure 8B). Notably, lenvatinib and regorafenib downregulated BUD31 expression more strongly than sorafenib (*p* < 0.01). Lenvatinib and regorafenib, but not sorafenib, significantly downregulated SF3A1 expression by more than 20% when compared with DMSO (*p* < 0.05, Figure 8B). Chen et al. (2015) demonstrated that SF3A1 is critical for spliceosome assembly and normal splicing events of pre-mRNA, which is related to the susceptibility of lung cancer and breast cancer (Chen et al., 2015). BUD31 protein depletion mainly increases intron retention. High expression of the splicing factor BUD31 is associated with earlier metastasis formation in triple-negative breast cancer and estrogen receptor-positive breast tumors (Koedoot et al., 2019). WBP11 is a novel protein in controlling centriole duplication and centrosome number (Park et al., 2020). The specific silencing of Prp19 gene expression enhances the apoptosis of HCC cells (Yin et al., 2014). UAP56 is a fundamental splicing factor necessary for facilitating U2 snRNP binding to pre-mRNA and it is important in the mRNA export from the nucleus to the cytoplasm (Fleckner et al., 1997). UAP56 can promote the proliferation of the CRC cells directly, and the tumor weight in the UAP56 knockdown model of nude mice is lower than the control group (Zhang et al., 2022).

The [U4/U6·U5] tri-snRNP (small nuclear ribonucleoprotein) has an important role in the generation of pre-mRNA splicing machinery. Four proteins (SPF30, Dib1, SART1 and SNRPF) were related to the machinery (Figure 8A). All of them, except for SNRPF, were significantly downregulated by sorafenib, lenvatinib and regorafenib, respectively (*p* < 0.05 and *p* < 0.01, Figure 8B). Sorafenib and regorafenib, but not lenvatinib, downregulated SNRPF expression significantly (*p* < 0.05), even though the SNRPF expression level in the lenvatinib group decreased to approximately 82.3% of that in DMSO group (Figure 8B). SPF30 (SMNrp) is detected in spliceosomal complexes containing the U2 snRNP, which may bind both the U2 snRNP and the [U4/U6⋅U5] tri-snRNP, thereby promoting the integration of the [U4/U6⋅U5] tri-snRNP into the pre-spliceosome (Meister et al., 2001). Specific silencing of SPF30 gene expression dramatically reduces the proliferation of human ovarian cancer A2780 cells (Giri et al., 2014). Plaschka et al. (2017) have reported that in the isolated U4/U6⋅U5 triple snRNP and B complex, Dib1 is adjacent to the U5 loop 1, occluding the U6 sequence required to position the pre-mRNA for splicing (Plaschka et al., 2017). Recent structural study suggests that Dib1 prevents the activation of premature spliceosome (Schreib et al., 2018). In nucleus of the most of proliferating cells, SART1 integrates the U4/U6.U5 tri-snRNP into the pre-spliceosome. Specific silencing of SART1 gene expression induces apoptosis of CRC cells (Allen et al., 2012). SNRPF was reported to be the most significantly upregulated protein among 83 overexpressed proteins in the cancer tissues of the patients with renal cell carcinoma (Sun et al., 2016). SNRPF participates in constituting a ring structure of Sm proteins (Smith proteins) (Grimm et al., 2013), and Sm proteins are involved in mRNA decapping and decay (Martens et al., 2017).

#### 3.4.4 mRNA surveillance signaling pathway

**FIGURE 9:**
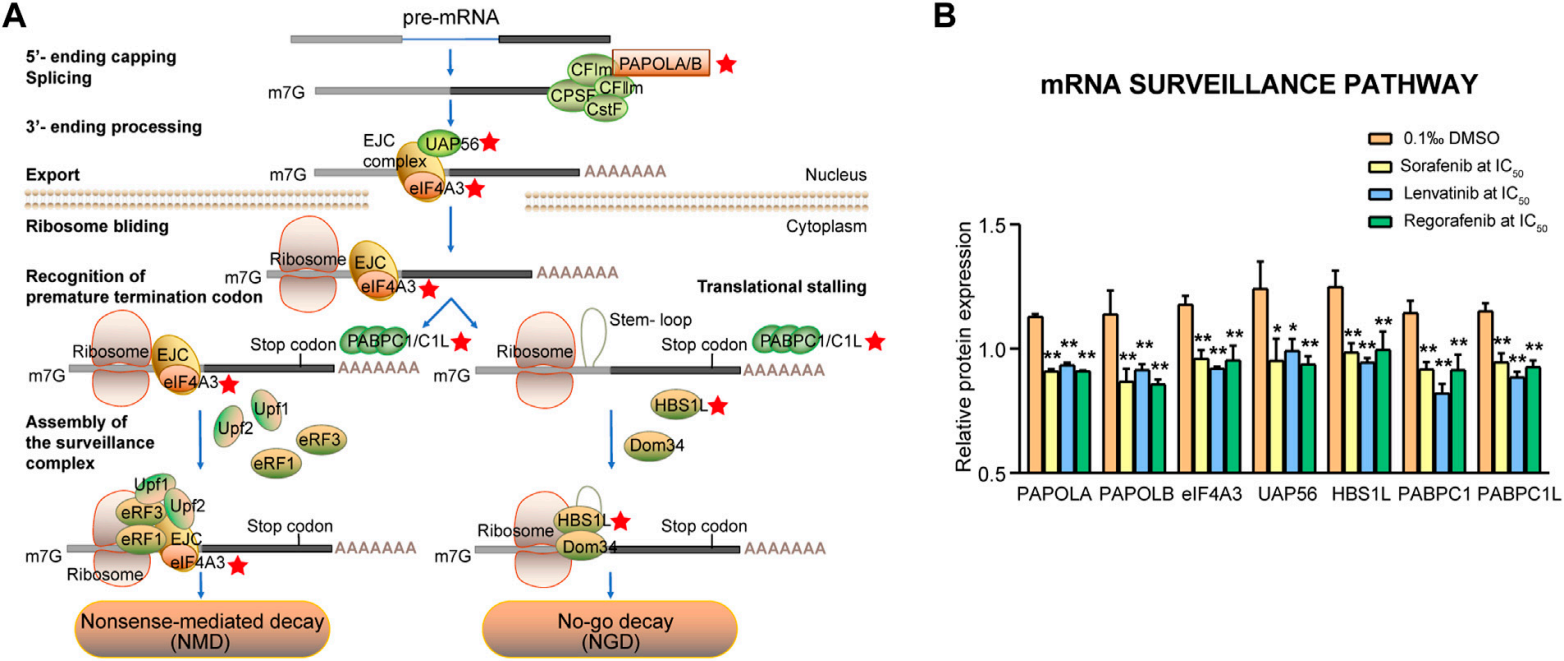
Commonly downregulated proteins involved in the mRNA surveillance pathway **(A)** and their relative expression **(B)** in Huh-7 cells treated by 72 h incubation with respective sorafenib, lenvatinib and regorafenib. Each bar presents mean ± SD (*n* = 3). The *p < 0.05 and **p < 0.01 versus DMSO. Star symbol in red color indicating the downregulation of the protein.

The mRNAs would be subject to the mRNA surveillance machinery if an mRNA is not spliced or spliced incorrectly. Therefore, mRNA surveillance pathways ensure accurate and efficient RNA processing by degrading the products of errors in RNA processing. In the present study, seven proteins were involved in the mRNA surveillance signaling pathway (Figure 9A), which were significantly downregulated by sorafenib, lenvatinib and regorafenib, respectively (*p* < 0.01, Figure 9B). The seven proteins were PAPOLA, PAPOLB, eIF4A3, UAP56, HBS1L, PABPC1 and PABPC1L, being responsible for the formation of mRNA long poly(A) tails, pre-mRNA splicing, mRNA nuclear export and mRNA degradation.

The canonical poly(A) polymerases (PAPs) exist in multiple isoforms and at least three forms of PAPOLA, PAPOLB and PAPOLG have been reported. PAPs are involved in the formation of long poly(A) tails at the 3′-end of eukaryotic mRNAs. As shown in Figure 9A, PAPOLA is necessary for pre-mRNA polyadenylation taking place in the nucleus, while PAPOLB may also be involved in the translation of specific mRNAs by cytoplasmic polyadenylation (Laishram, 2014). The exon junction complex (EJC), a key determinant of mRNA metabolism, is assembled on mRNAs, and the eIF4A3 is responsible for anchoring of EJC correctly on mRNA during the splicing process (Ballut et al., 2005). Luo et al. (2001) indicated that UAP56 functions in coupling the splicing and export machineries by recruiting Aly to the spliced mRNP (Luo et al., 2001). No-go decay proteins of HBS1L and PELO are the translation quality control proteins, and the former has been implicated in the recycling of inactive ribosomes (O'Connell et al., 2019). Polyadenylate binding proteins (PABPs) are divided into the nuclear PABPN1 and the cytoplasmic PABPCs. The latter, including PABPC1, PABPC1L and others, are involved in many functions of the translation, mRNA decay, and adjusting mRNA deadenylation rate. In the absence of PABPC1, eukaryotic polypeptide release factor (eRF3a) recruits the nonsense-mediated decay (NMD) factor UPF1 to the terminating ribosome, thus triggering mRNA degradation (Fatscher et al., 2014). PABPC1 expression is higher in HCC tissues and promotes entry into the S phase in cell cycle (Zhang et al., 2015). After the depletion of PABPC1L in the human colon adenocarcinoma HT-29 cells, the cell proliferative, invasive and migratory capacities are inhibited significantly (Wu et al., 2019).

#### 3.4.5 Signaling pathways involved in nucleotide excision repair and DNA replication

**FIGURE 10:**
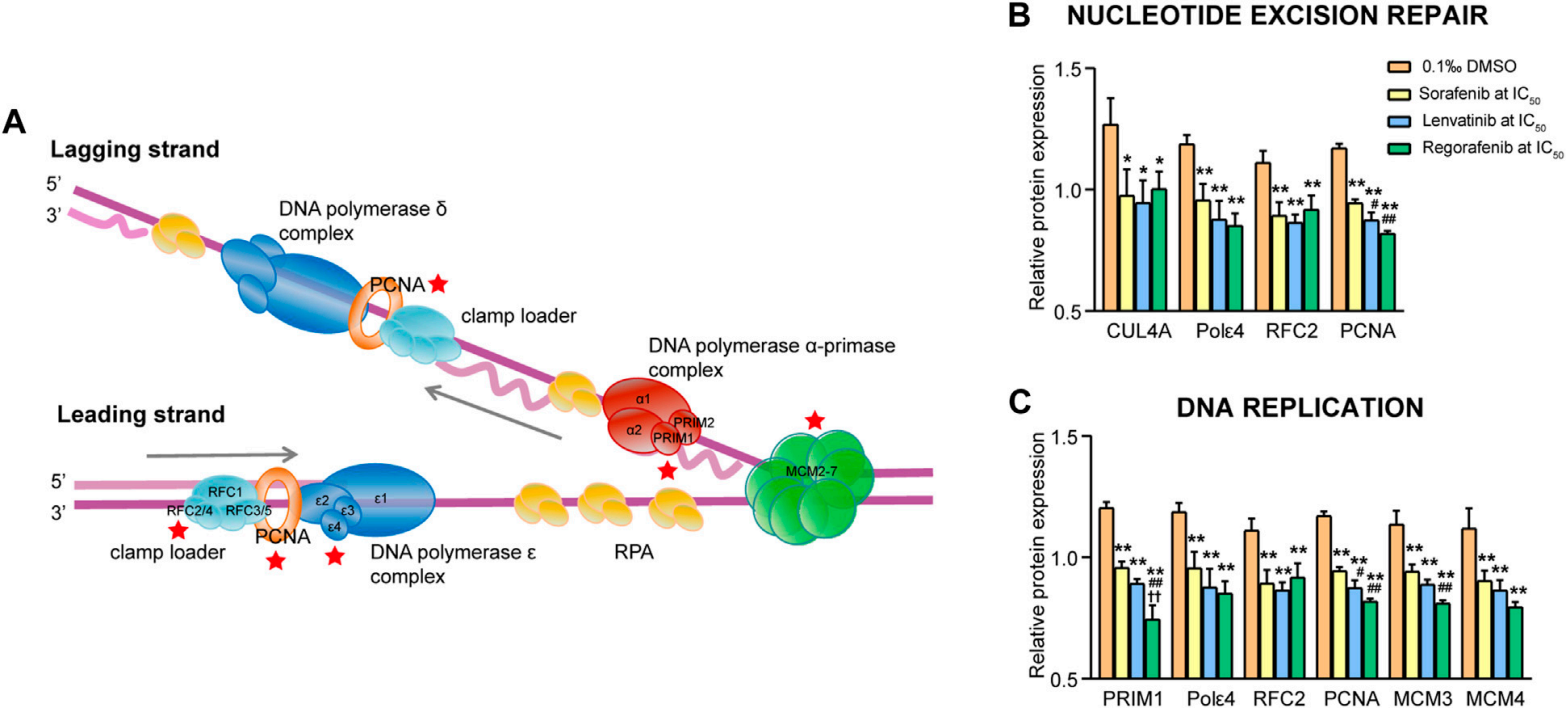
Commonly downregulated proteins involved in the nucleotide excision repair and DNA replication pathway **(A)** and their relative expression (**B** and **C)** in Huh-7 cells treated by 72 h incubation with respective sorafenib, lenvatinib and regorafenib. Each bar presents mean ± SD (*n* = 3). The *p < 0.05 and **p < 0.01 versus DMSO; ^#^ *p* < 0.05 and ^##^ *p* < 0.01 versus sorafenib; ^††^ *p* < 0.01 versus lenvatinib. Star symbol in red color indicating the downregulation of the protein.

Mammalian nucleotide excision repair (NER) includes two sub-pathways: the global genome repair pathway eliminating lesions throughout the genome, and the transcription-coupled repair pathway selectively repairing lesions in transcribed DNA strands. In the present study, four downregulated proteins (CUL4A, Pol ε4, RFC2 and PCNA) were related to the NER processes, and six downregulated proteins (PRIM1, Pol ε4, RFC2, PCNA, MCM3 and MCM4) were related to the DNA replication processes (Figure 10A). Since the most proteins in the two pathways were overlapped, we analyzed them together. The four proteins in the NER pathway were significantly downregulated by sorafenib, lenvatinib and regorafenib, respectively (*p* < 0.05 and *p* < 0.01, Figure 10B). In the DNA replication pathway, protein PRIM1 was significantly downregulated by the three inhibitors, and the PRIM1 expression level in the regorafenib group decreased to 61.9%, 77.8% and 83.6% of that in DMSO, sorafenib and lenvatinib groups, respectively (*p* < 0.01, Figure 10C).

CUL4A, a crucial component of CUL4A-based ubiquitin ligase, regulates proteolysis of DDB2 (a sensor of DNA damage) degradation at DNA damage sites, and facilitates the efficient recruitment of XPC protein (an essential damage recognition protein) (El-Mahdy et al., 2006). Moreover, knockdown of CUL4A inhibits the proliferation of HepG2 cells, accompanied by S-phase reduction (Pan et al., 2015). DNA replication in eukaryotic cells requires at least three B-family DNA polymerases, including the initiation reaction by DNA polymerase (Pol) α, followed by Pol ε and Pol δ functioned on the leading and lagging strands, respectively (Figure 10A). Both Pol ε3 and Pol ε4 localize to the nucleus and a normal localization of Pol ε4 is dependent on expression of Pol ε3 (Spiga and D'Urso, 2004). The elevated Pol ε expression significantly correlates with shorter overall survival of patients with clear cell renal cell carcinoma (Wu et al., 2021). Replication factor C (RFC) including its RFC2 subunit is involved in many processes of the DNA replication, DNA replication checkpoint and DNA repairing (Qiu et al., 2021). Ji et al. (2021) have demonstrated that a higher RFC2 expression is significantly correlated with shorter overall survival and disease-free survival in patients with liver cancer, and knockdown of RFC2 reduces the proliferation and migration of the HepG2 cells (Ji et al., 2021). Boehm et al. (2016) have reported that PCNA plays critical roles in many aspects of DNA replication and replication-associated processes, including break-induced replication, mismatch repair and chromatin assembly (Boehm et al., 2016). PRIM1 containing two subunits synthesizes the RNA primer for the discontinuous DNA replication (Lee et al., 2019). Silencing of PRIM1 gene expression in human hepatic carcinoma cells significantly inhibits the cell proliferation and increases the cell apoptosis (Jiang et al., 2020). The relationship between DNA replication and MCM3 or MCM4 has been described above.

## 4 Summary and conclusion

In summary, the present study for the first time performed a direct comparison of the cell cycle arrest and apoptosis induction caused by sorafenib, regorafenib and lenvatinib at respective IC_50_ as well as their pharmacological interventions for influencing whole cell proteomics in Huh-7 cells. While the three multi-RTK inhibitors all drove the remaining surviving Huh-7 cells into a G_0_/G_1_ arrest, lenvatinib and regorafenib were much more effective than sorafenib. Lenvatinib produced a much stronger induction of Huh-7 cells into early apoptosis than sorafenib and regorafenib, but necrotic cell proportion induced by regorafenib was 2.4 times as large as that by lenvatinib. The proteomic study revealed 419 proteins downregulated commonly by the three inhibitors. KEGG pathway analysis of the downregulated proteins revealed the top six signaling pathways of the spliceosome, DNA replication, cell cycle, mRNA surveillance, P53 and nucleotide excision repair, all of which were directly related to the effects of the three inhibitors on cell cycle and cell apoptosis. More importantly, the downregulation of PCNA, Cyclin B1, BCL-xL, TSP1, BUD31, SF3A1 and Mad2 proteins involved mainly in the cell cycle, spliceosome and P53 signaling pathways by lenvatinib and regorafenib was much stronger than that by sorafenib, and most of the proteins in P53 signaling pathway were downregulated with the two inhibitors by more than 36% at least. These findings probably provide stronger evidence to support the combination therapy of immune checkpoint inhibitor with lenvatinib or regorafenib better than sorafenib.

Recently, Leone et al. (2021) indicated that the tumor microenvironment in HCC is strongly immunosuppressive and new treatment approaches for HCC immune escape are necessary. Therefore, immunotherapy based on the use of immune checkpoint inhibitors, as single agents or in combination with multi-RTK inhibitors, anti-angiogenic drugs, chemotherapeutic agents, and locoregional therapies, offers great promise in the treatment of HCC (Leone et al., 2021). We need to further investigate whether the findings of the present study can be confirmed in the tumor-bearing mouse model, especially whether the regulated signal pathways by the three multi-RTK inhibitors are affected by the new first-line immune checkpoint inhibitors such as anti-PD-L1 inhibitor durvalumab, anti-PD1 monoclonal antibody pembrolizumab or anti–CTLA-4 antibody tremelimumab. In conclusion, lenvatinib and regorafenib have much stronger potency against Huh-7 cell proliferation than sorafenib depending on their more potent effects on cell cycle arrest and apoptosis induction. The underling mechanism may be at least due to the 33 downregulated proteins involved in the signal pathways centralizing cell cycle, p53 and DNA synthesis based on the proteomic study.

## Data Availability

The datasets presented in this study can be found in online repositories. The names of the repository/repositories and accession number(s) can be found below: ProteomeXchange Consortium *via* the PRIDE partner repository with the dataset identifier PXD034073.
