# Supplementary figures and images for: Cell-cycle and apoptosis related and proteomics-based signaling pathways of human hepatoma Huh-7 cells treated by three currently used multi-RTK inhibitors
Source: Front Pharmacol. 2022 Aug 22;13:944893. doi: 10.3389/fphar.2022.944893 (PMC9444053; doi:10.3389/fphar.2022.944893)

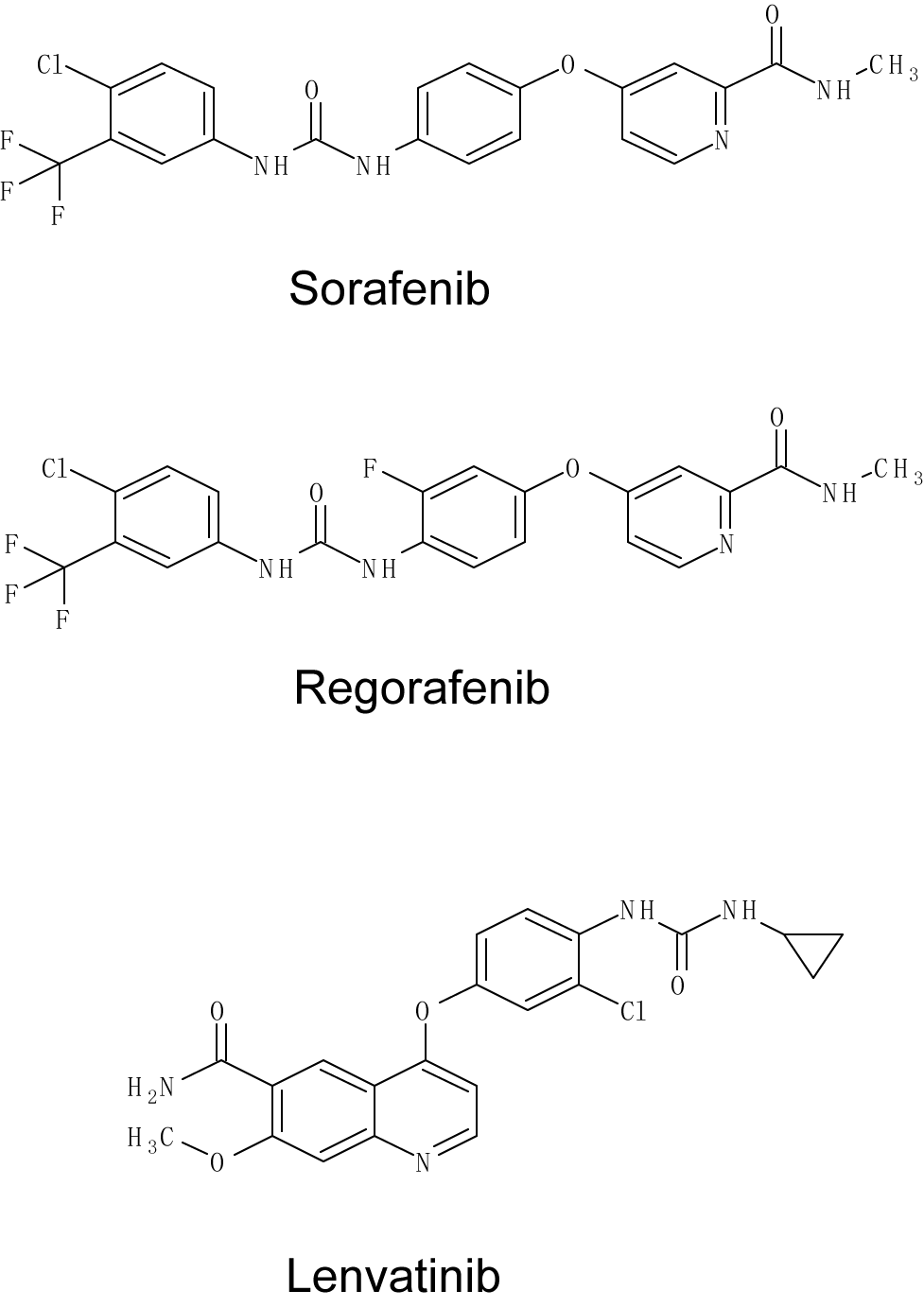

Supplement: Supplementary file 1 [file Image3.TIF]

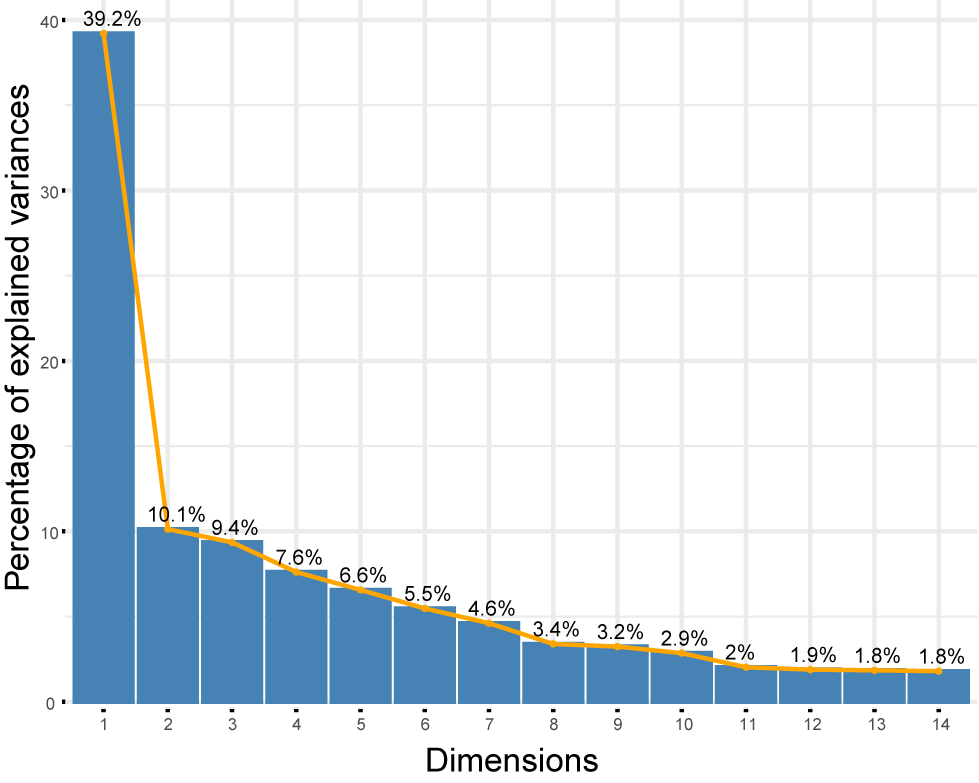

Supplement: Supplementary file 2 [file Image2.TIF]

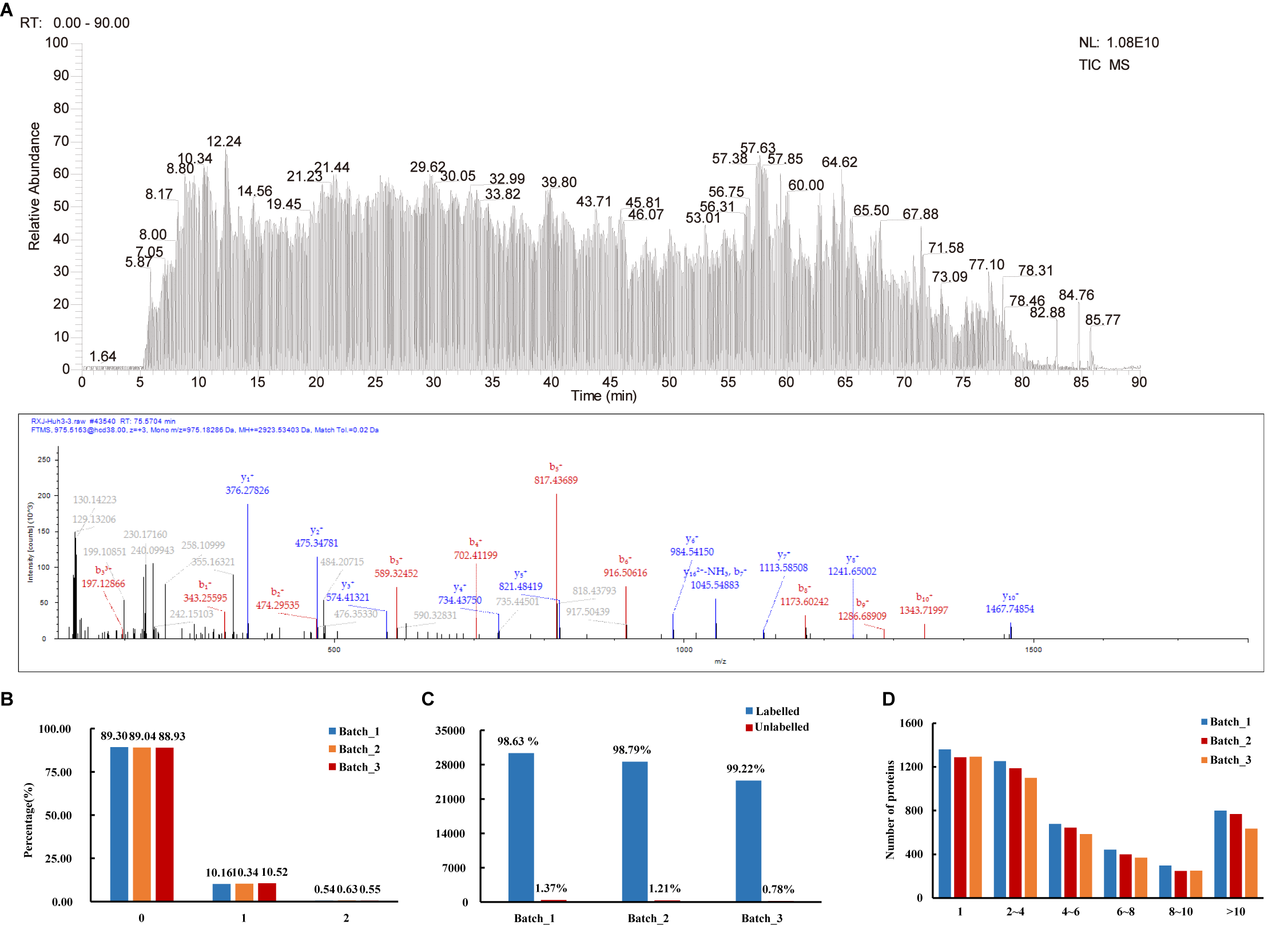

Supplement: Supplementary file 3 [file Image1.TIF]
